# Emerging Leptospirosis, North India

**DOI:** 10.3201/eid0812.020052

**Published:** 2002-12

**Authors:** Rama Chaudhry, M.M. Premlatha, Srujana Mohanty, Benu Dhawan, Kumar Kirti Singh, A.B Dey

**Affiliations:** * India Institute of Medical Sciences, New Delhi, India

**To the Editor**: We read with interest the article, The Changing Epidemiology of Leptospirosis in Israel, published in volume 7, no. 6 (1). Leptospirosis, a septicemic zoonosis with multisystemic involvement, is caused by the pathogenic strains of *Leptospira interrogans*. Rural farm workers are at high risk for leptospirosis, and it can be a significant public health problem when water and food safety are not ensured. Several epidemics of leptospirosis have occurred on Andaman and Nicobar islands and in southern and western parts of India during the past century (2). The organism has been detected in farm animals in many parts of the country (3); however, human infections have been more or less localized. In 1998, researchers warned that, unless adequate public health measures were initiated, large leptospirosis epidemics were possible in areas where the disease had not been previously reported (4). In addition, they recommended improving clinical diagnosis and conducting systematic epidemiologic studies to control of the disease (4).

The true incidence of human leptospirosis in northern India is not known either because of a lack of awareness on the part of the treating physicians or the lack of diagnostic techniques. In 1966, human leptospirosis was reported in Delhi, a state in northern India (5). In a 1966 study (5), sera from persons with pyrexia and jaundice were tested by the agglutination lysis test for leptospiral antibodies. Of 93 serum specimens from persons with pyrexia cases, 3 were positive (1 with *L. icterohemorrhagica* and two with *L. canicola*); of 43 serum specimens from persons with jaundice, 3 were positive (2 with *L. icterohemorrhagica* and 1 with *L. icterohemorrhagica* and *L. pomona*). No other study on leptospirosis has been done in the region, and no data are available concerning the problem.

To assess the current status of transmission in Delhi and its adjoining areas, we conducted a systematic study for the diagnosis of leptospirosis in our hospital from April 2000 to March 2001; case definition criteria suggested in a previous study (4) were used. A case was defined as a person with fever, headache, and myalgia and more than two of the following symptoms: jaundice, oliguria, respiratory symptoms (cough, hemoptysis, and breathlessness), hemorrhagic manifestations (hematemesis, bleeding gums, and subconjunctival hemorrhage), and signs of meningeal irritation and convulsion. Seventy-five patients (44 male patients; 3–73 years of age) satisfied the inclusion criteria. In addition to clinical evaluation and assessment for other diseases, leptospirosis was investigated by the following laboratory methods: isolation of *Leptospira interrogans*, direct visualization of the organism under dark-field microscopy, and enzyme-linked immunosorbent assay (ELISA) for *Leptospira* immunoglobulin (Ig) M antibody (Serion Immunodiagnostica GmbH, Würzburg, Germany). Per manufacturer’s specifications, the sensitivity, specificity, positive predictive value, and negative predictive value of this kit are 96%, 97%, 90%, and 99%, respectively). All blood samples were sent to the *Leptospira* referral laboratory at the Indian Veterinary Research Institute, Izzatnagar, for microscopic agglutination test (MAT). Eight serovars of *L. interrogans* (*australis, autumnalis*, *pomona, sejroe, tarassovi, icterohaemorrhagica, hebdomadis,* and *patoc*) were tested, and a agglutination titer of more than 1:100 was considered positive. All patients were treated empirically with broad-spectrum antibiotics as well as specific drugs according to the results of investigations.

Thirty-two patients (42.6%) had a positive ELISA test for *Leptospira* IgM antibody. The results of MAT were positive in 21 (65.6%) of the 32 ELISA-positive serum samples. Serum specimens from 11 patients reacted with a single serovar, and specimens from 10 patients reacted with more than one serovar. Among the pathogenic species, *Leptospira* antibodies were detectable by MAT predominantly against *L. sejroe* (7 of 21), followed by *L. icterohaemorrhagica* (6 of 21), *L. hebdomadis* (4 of 21), and *L. tarassovi* (4 of 21). *Leptospira* antibodies were also detectable against *L. autumnalis* (3 of 21), *L. australis* (2 of 21), and *L. pomona* (1 of 21). Against *L. patoc*, MAT could detect antibodies in six samples. The organism could not be isolated in culture or visualized under dark-field microscopy in any of the specimens. Of the 43 case-patients with ELISA-negative specimens, alternative diagnoses were established for 40 on the basis of various laboratory investigations. In five of the case-patients with ELISA-positive specimens, coinfection with other pathogens was detected, including *Salmonella typhi* (one case) by a positive Widal test, hepatitis C virus by positive ELISA (two cases), and *Plasmodium falciparum* (two cases) by a positive smear. Five patients, including three who were ELISA positive for *Leptospira*, died. The highest number of ELISA-positive serum samples (21 of 32) were obtained in August and September 2000, suggesting an epidemic.

Epidemiologic investigation of leptospirosis is often hampered by the difficulty of making a definitive microbiologic diagnosis. Isolation of leptospira from clinical samples provides a definitive diagnosis; however, the value of culture is limited because samples have to be collected before the administration of antibiotics, and culturing requires prolonged incubation. Demonstration of typical motility of leptospira under dark-ground illumination in clinical samples, though helpful in early diagnosis, has low sensitivity and depends on the technician’s opinion. Measurement of IgM antibodies against *Leptospira* by ELISA has emerged as a reliable diagnostic test with good specificity and sensitivity (6). The probability of achieving a positive serologic test increases with the duration of disease, and good correlation between results of MAT and ELISA has been reported by Cumberland et al. (7). MAT has emerged as a dependable diagnostic tool for leptospirosis (next to isolation) by providing serovar specific diagnosis. However, a large number of serovars of *L. interrogans* exist, and maintaining large numbers of organisms for MAT is difficult for most laboratories. Moreover, MAT may fail to detect antibodies when specific serovars are not used. In this study, the ELISA-positive samples, for which MAT results were negative, may have been caused by infection with serovars other than those used in this study. Because of the problems with methods, leptospirosis is grossly underdiagnosed.

*Leptospira* organisms require humid weather for their survival. Rodents and domestic animals (i.e., cattle and dogs) harbor leptospires and shed the bacteria in urine; they may disseminate the organism in the rain and drinking water sources. Humans frequently come into contact with contaminated water during floods; the number of cases is higher during and after heavy rainfalls. We found that the peak incidence of the disease was during August and September, the monsoon season, which may explain the high incidence of seropositivity during this period. Though the organism has been detected in farm animals in northern India, human leptospirosis has not been considered a major public health problem, probably because transmission is low in arid weather conditions. As a result of 13 consecutive monsoons of above-average strength in India, changes in the environment may be promoting the transmission of this organism. Recently, two other regions in northern India, Chandigarh (8) and Varanasi (9), have reported a *Leptospira* seroprevalance rate of 8.8% and 21.74%, respectively.

Our study supports the warning from other researchers regarding the threat of leptospirosis in areas such as northern India. Preventive measures should be initiated and rapid and definitive diagnostic tests must be developed.
